# Deciphering the global research trends and significance of moral intelligence via bibliometric analysis

**DOI:** 10.3389/fpsyg.2024.1425341

**Published:** 2024-09-02

**Authors:** Dipanjan Bagchi, Akancha Srivastava, Bhawna Tushir

**Affiliations:** ^1^Department of Psychology, CHRIST (Deemed to be University) Delhi NCR, Delhi, India; ^2^Jindal School of Psychology and Counselling, O.P. Jindal Global University, Sonipat, Haryana, India

**Keywords:** moral intelligence, moral injuries, moral measurement, bibliometric and network analysis, VOS view analysis, biblioshiny analysis, moral psychology

## Abstract

**Introduction:**

Moral Intelligence (MI) as a concept has gained importance in recent years due to its wide applicability in individual, organizational, and clinical settings or even policy making. The present study employed Bibliometric analysis to understand the emerging topics associated with MI and its global research trend. This paper’s primary aim was (i) to explore the temporal and geographic growth trends of the research publication on MI. (ii) to identify the most prolific countries, institutions, and authors, working on MI, (iii) to identify the most frequent terminologies, (iv) to explore research topics and to provide insight into potential collaborations and future directions, and (v) to explore the significance of the concept of moral intelligence.

**Method:**

Bibliometric analysis was used to understand the emerging topics associated with MI and its global research trend using the SCOPUS database. VOS viewer and R were employed to analyze the result. Through the analysis conducted, the development of the construct over time was analyzed.

**Results:**

Results have shown that Iran and the United States and these two combined account for 53.16% of the total country-wise publications. Switzerland has the highest number of Multi-county publications. Authors from Iran and Switzerland have the most number of publications. Emerging topics like decision-making, machine ethics, moral agents, artificial ethics, co-evolution of human and artificial moral agents, green purchase intention etc were identified.

**Discussion:**

The application of MI in organisational decision-making, education policy, artificial intelligence and measurement of moral intelligence are important areas of application as per the results. Research interest in MI is projected to increase according to the results delineated in this article.

## Introduction

Moral intelligence (MI) is an important area of study, as the fast-paced, interconnected world has created unprecedented moral challenges for individuals, institutions, and societies. Researchers are increasingly interested in understanding how moral intelligence can influence decision-making and behavior in various fields, including education, healthcare, and organizational settings. MI intersects with various disciplines such as psychology, ethics, education, and artificial intelligence. This interdisciplinary nature allows for diverse perspectives and methodologies, making it a rich topic for exploration and responsible innovations. Research indicates moral intelligence is a multifaceted construct involving cognitive and emotional capacities. It is influenced by culture, education, and organizational environment (Bentahila et al., 2021). Enhancing moral intelligence is crucial for developing ethical decision-making and prosocial behaviors. The growing body of research on moral intelligence underscores its importance in navigating the complex moral landscape of the modern world. Continued efforts to understand and cultivate moral intelligence can be leveraged to address issues involving individuals, organizations, and societies effectively. Therefore, comprehending global research trends is essential for guiding researchers in making informed and strategic research decisions.

The concept of Moral Intelligence (MI) has undergone significant evolution in recent years. Initially conceptualized by Howard Gardner, MI emerged as part of his broader theory of Multiple Intelligences. Gardner (1983) identified eight distinct intelligences and later considered additional candidates, including social, emotional, spiritual, existential, and moral intelligence (Gardner 1998a,b,^1999^). Despite the growing prominence of social and emotional intelligence, the concept of Moral Intelligence has remained relatively underexplored. Gardner (1999b), argued against classifying MI as a separate intelligence. He cited three main reasons: cultural specificity, complexity, and the diversity of moral judgments (Gardner, 2007). Despite Gardner’s reservations, other researchers have continued to explore the potential of moral skills as a distinct form of intelligence, diverging from Gardner’s initial framework. This discussion will examine the evolving definitions of Moral Intelligence as proposed by various scholars over time.

### Recent advancement in MI conceptualization

MI can be defined as a form of ability concerned not with the intention but with selecting appropriate action in moral situations while dealing with their own emotional and cognitive dilemma (Gardner 1983). While, Borba (2001) has described it as the capacity to recognize others’ pain and restrain from acting cruelly, controlling and delaying gratification, considering different perspectives before judgment, tolerating differences, understanding unethical choices, empathizing, opposing injustice and treating others with compassion (Borba, 2001). Lennick and Kiel (2005) defined moral intelligence as ‘the mental capacity to determine how universal human principles should be applied to our personal values, goals, and actions’. They have identified 9 competencies. Out of these nine competencies, integrity has four competencies, responsibility has three and forgiveness has two. Tanner and Christen (2014), defined a morally intelligent person as someone endowed with a desire to strive for moral goals and to use moral principles and self-regulatory skills to do what is good for society, other humans, or other human-nonhuman beings. They proposed four process structures of Moral Intelligence: Moral Sensitivity, Moral Competence, Moral Problem solving and Moral Resoluteness.

### Changing nature of moral intelligence

A closer examination of all the definitions of Moral Intelligence reveals that this concept has been broadening with the progress of time. The potential dynamic nature of MI had been tapped first by Gardner. According to him, the concept is limited to choosing an appropriate action while dealing with emotional and cognitive dilemmas (Gardner 1983). However, omitting the intention part has major implications. Understanding the intentions of others is an essential feature of MI. Other scholars, such as Borba (2001), delineated the attributes of a morally intelligent individual. While significant, this conceptualization remains idealistic. Even if individuals conform to Borba’s criteria, establishing the measure’s validity presents considerable methodological challenges. Individuals, at some juncture, are inclined to partake in behavior that may be deemed morally ambiguous; consequently, this definition inadequately encapsulates the true essence of moral integrity.

Lennick and Kiel (2005) proposed a definition of moral intelligence emphasizing the traits that make up this construct. Integrity, responsibility, forgiveness and compassion were identified as the main compentencies. One competency related to integrity is telling the truth, but how much of it is a sign of being morally intelligent has not been delineated. Real-life situations are more complex than adhering to certain values and principles. Lastly, Tanner and Christian’s definition took a process-based approach and included the concept of moral resoluteness among others. It refers to the ability to maintain consistent and persistent adherence to moral standards when faced with danger and threats to self and the stamina to pursue moral actions (Tanner and Christen, 2014). The components of moral resoluteness are resistance, courage, consistency and perseverance (Blasi, 2005; Sekerka and Bagozzi, 2007). They did try to capture the essence of daily life in their definition but it needs further deliberation as to the scope and boundaries of this concept.

### The current state of knowledge

The above definition shows the changing nature of MI and its potential dynamicity. It is evident from the literature there have been various approaches to defining moral intelligence each adding to the uniqueness of MI. One approach to classifying the different approaches can be abilities and trait-based models. Gardner (1983) adopted an ability-based approach while defining Moral Intelligence. While it was conceptualized in terms of a trait-based approach like Borba and Lenick and Kiel. In this regard, the most recent definition of MI by Tanner and Christen (2014) is also a trait-based approach. However, no model combining the two approaches has been proposed yet. It is important to note that combination of a trait-based and ability-based approach can pave the way forward to make MI a formidable concept in moral psychology. In this regard, it should be noted that this approach has been adopted in Emotional Intelligence as well (O’Connor et al., 2019).

**Figure 1:**
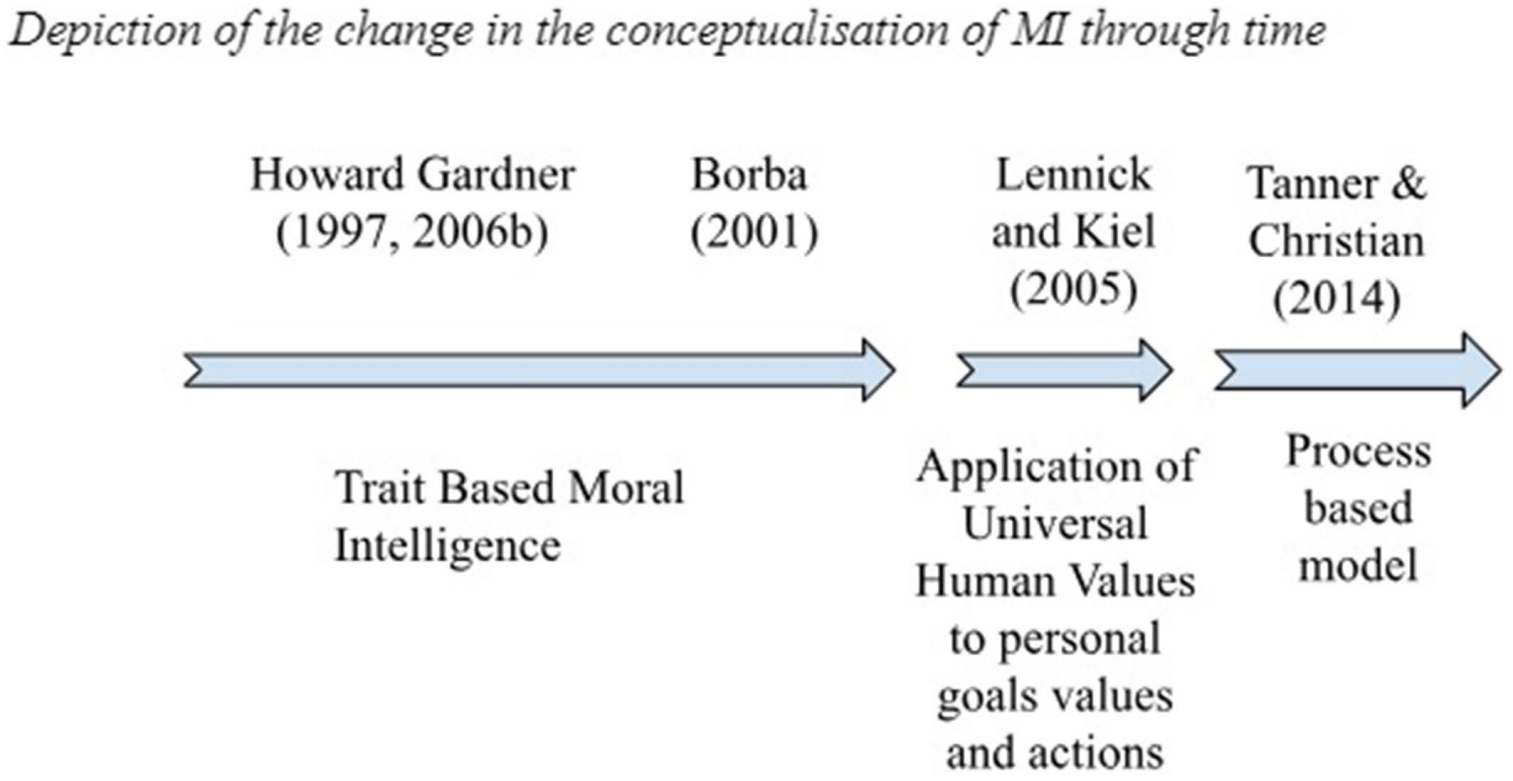
Depiction of the change in the conceptualization of MI through time.

It is important to point out here that theory development is another aspect of MI which has not received much attention from researchers. Tanner and Christen (2014) proposed a theoretical model of MI that comprises four process structures, apart from that the research literature does not shed much light on the dimensions of MI. A concrete theoretical base is a prerequisite for proposing a model. Hence in this article, a Bibliometric analytic approach was adopted to find out the various scattered literature available in the research domain that can ultimately be unified by researchers. Here we see the timeline of the definition and try to evaluate the changing nature of the concept of Moral Intelligence (Figure 1).

### Relevance of MI in everyday life

Moral Intelligence as a construct has largely been ignored for a long time but is of great importance as it is essential in guiding our behavior in day-to-day life. MI can be an integral part of well-being and progress (Boss, 1994).

#### Crime rate and training on MI

According to the National Crime Records Bureau, Government of India, in 2020 a total of 66,01,285 cognizable crimes were registered. It shows an increase of 14,45,127 (28.0%) than that was registered in the year 2019. Records were unavailable for 2021 and 2022 (National Crime Records Bureau, 2021). Around 76% of the juvenile cases registered were caused by adolescents with an age range of 16–18 years. It has been stated in this document that crime is a result of the inability to develop a strong sense of right and wrong. As a result, people recourse to criminal activities to satisfy their needs and desires (National Crime Records Bureau, 2021). Hence it becomes incumbent on the parents and educational institutions to sensitize children on moral issues and make them more morally sensitive. Training needs to be imparted not only on moral sensitivity but also on solving real-life moral dilemmas, which involve children making decisions while safeguarding their moral values to the best of their abilities.

#### Moral intelligence and moral injury

The importance of MI in an individual’s well-being can be understood in the context of another evolving concept- Moral Injury. It is defined as, ‘the distressing psychological, behavioral, social, and sometimes spiritual aftermath of exposure to such events’ (Norman and Maguen 2020). It may occur in response to witnessing or acting in a manner that is opposed to one’s values and moral beliefs. It can lead to severe psychological distress and has been differentiated from PTSD, although symptoms can be very similar. According to the current literature, a person meeting the criteria of moral injury may not be meeting the same for PTSD (Bryan et al., 2018). Moral injury is associated with greater PTSD and varying levels of depressive symptoms. It has been associated with increased suicidal intent and behavior (Bryan et al., 2018; Currier et al., 2019; Koenig et al., 2018). MI deals with decisions and their execution in real-life settings, while moral injury deals with psychological distress that results from a breach of personal values. Likewise, everyday life situations also pose threats of moral injury which can be navigated by providing training on moral intelligence.

#### Clinical disorders and MI

MI as a concept has the potential to add new ways of looking at the existing knowledge in clinical psychology, be it research or practice. Its importance is also understood when there is an aberration in the typical development. A child who met with a prefrontal cortex injury at 15 months of age was found to be unempathetic, stealing, lying, and unresponsive to verbal instructions and punishment and showing disruptive behavior as a teenager (Anderson 1999). In another case, symptoms including stealing without remorse were found (Anderson, 1999). Clinical disorders like antisocial personality disorder, are associated with a lack of empathy, and engaging in criminal behavior without remorse and are associated with the second stage of Kohlberg’s model of morality. It has been found that children with conduct disorder show a developmental delay in moral judgment (Gibbs, 2003).

In clinical practice, training on the development of moral intelligence can pave the way for coping with difficult life situations and maintaining one’s mental health. Also, certain clinical disorders like antisocial personality disorder and conduct disorders, are associated with a lack of empathy, and engaging in criminal behavior without remorse and are associated with the second stage of Kohlberg’s model of morality. It has been found that children with conduct disorder show a developmental delay in moral judgment (Gibbs, 2003). Improving their Moral Intelligence is important. The above facts points out the value that MI can add to clinical research and practice. More research is needed to determine the effectiveness of Moral intelligence training in overcoming these problems. As it is clear from the above discussion that MI as a construct can explain the whole spectrum of human behavior in the context of morality, it is important to look into the research advances we have made to date. Scientific literature dedicated to Moral Intelligence is slowly but steadily increasing. Hence, it is important to understand the state of the current intellectual structure (Donthu et al., 2021). There is a dearth of work in terms of building the concept of MI and its dimensions. With this aim in mind bibliometric analysis was used as the scope of this paper is broad and it helps in understanding the research terrain on this topic. Also, this research is not confining the application of MI to a specific discipline. It will help to understand this emerging field and guide researchers as to the extent of work that has been done. SCOPUS database was selected for collecting the data required for this study. As SCOPUS is regarded as the largest abstract and citation database for peer-reviewed journals it was selected over Web of Science (Donthu et al., 2021).

In this research, Bibliometric analysis has been used to gain insights into the current literature on MI. It was chosen in this paper as it helps in deciphering and mapping the scientific knowledge of a topic of interest. It helps in working with a pool of unstructured data and finding patterns in it. A Bibliometric study aims to give a one-stop overview of the scientific literature related to a topic of interest, search for knowledge gaps, and find new ideas for scientific investigation. Hence, it was adopted in the study to make the research landscape of MI clear for researchers and scholars.

The aim of this paper in this study is to (i) explore the temporal and geographic growth trends of the research publication on MI. (ii) to identify the most prolific countries, institutions and authors, working on MI, (iii) to identify the most frequent terminologies, (iv) to research topics and to provide insight into potential collaborations and future directions and (v) to explore the significance of the concept of moral intelligence.

## Methods

### Data source and search strategy

The first step in the bibliometric analysis is to decide which database to use. For the present study, the Scopus database was selected. Data mining was conducted between June 9 and 14 April 2024 using the SCOPUS database. Articles relating to Moral Intelligence were searched on the SCOPUS database as it relate to the main theme of the paper. The search string used was TITLE-ABS-KEY (“Moral Intelligence*”). Quotation marks were used as it helps to retrieve the documents with the exact words from the Scopus database. This search strategy helped in retrieving documents that have “Moral Intelligence” mentioned together as opposed to documents where the words “Moral” and “Intelligence” are present separately in different sections of the document.

This search yielded 94 documents. The oldest article included in this data dates back to 1994 by Boss titled The Autonomy of Moral Intelligence and the most recent one was published in 2022. Glänzel (2013) suggested a minimum number of data for Bibliometric analysis to be 50 and Seglen (1994) suggested it to be between 50 and 100 articles. Hence we decided to proceed with the Bibliometric study.

### Eligibility criteria

As the scope of this study was broad, original articles, book chapters, and conference papers were included in the analysis. Conference papers were included to identify the current research trends. However certain documents were excluded from the analysis meeting the following criteria: a translated version of an article or review, editorials, letters and short surveys, and any duplicate document.

### Selection and data management

Both authors of the paper independently assessed the articles based on the inclusion and exclusion criteria mentioned above. In case of disagreement over the selection of an article, a third independent reviewer was consulted helping to maintain objectivity in the selection process. Authors with similar surnames were differentiated by using a Scopus author identifier that matches authorship with groups of documents. Scopus often classifies similar data under different heads such as book chapters, and books. These categories were considered as one entity and reclassified as books (Table 1).

**Table 1:** Details of the Scopus database used in the present study.

Indicators	Description
Database source	Scopus
Record volume	104
Publication type included	94 Articles (*n* = 73), Book (*n* = 2), Book Chapters (*n* = 14) Conference Paper (*n* = 4)
Publication type excluded	10 Conference Review (*n* = 1) Editorial (*n* = 1) Letter (*n* = 1) Short Survey (*n* = 1) Review (*n* = 6)
Selection strategy	TITLE-ABS-KEY (“Moral Intelligence*”)
Record content	Publication type, title, authorship, abstract, keywords, institution, country/region, journal, and citation frequency
Retrieval time	June 9 and 14 April 2024

### Analysis

The analysis part has been divided into performance and science mapping. Below we have given an account of its different aspects that will be discussed in this paper.

#### Performance analysis

Performance analysis is the description of the contribution of the research constituents (Cobo et al., 2011; Ramos-Rodrígue and Ruíz-Navarro, 2004). Biblioshiny from the Bibliometrics package of R was used to get insight into the research constituents on the topics. Moral Intelligence is the central theme of this paper and the search result was analyzed based on the number of documents by year, affiliations, country, document type, subject area and journals. Also, authors’ production over time, authors’ impact, and most relevant authors. Data on single-country publications and multiple-country publications were discussed, shading some insight into the intra-country as well as inter-country collaboration. It will be used to also understand the most locally cited documents and globally cited documents.

#### Science mapping

Science mapping focuses on the relationship between research constituents (Donthu et al., 2021). Citation, bibliographical information and author keyword information were exported to VOSviewer (version 1.6.18, Center for Science and Technology Studies, Leiden University, The Netherlands). It is software for network visualizing network maps. This type of analysis includes co-authorship and it was analyzed in terms of (units) authors, organizations and countries. The link strength in this analysis will indicate the number of collaborations between authors, organizations and countries. Another type of analysis used was the co-occurrence of author keywords where the link strength between keywords will be indicative of the number of keywords that occur together in different publications.

**Analysis of co-occurrence.** The analysis of the co-occurrence of keywords only included all the author keywords. Scopus-indexed keywords were excluded to gain more insight into the work done by the researchers in this field. A total of 210 authors’ indexed keywords were included in the analysis. Before the analysis with VOSviewer, all the keywords were scanned for repetition and congeneric words. In that case, those keywords were removed from the analysis. For example, ‘moral competencies’ and ‘nurse’ were repeated twice in the list of keywords. In that case, they were counted as one. Similarly, in the case of congeneric phrases like ‘responsibility’, ‘responsibilities’, ‘competence’ and ‘competencies’, only one was selected. In VOSviewer the minimus co-occurrence of keywords was set to 1 to identify emerging themes of study.

## Results and discussion

### Publication output and growth of research interest

**Figure 2:**
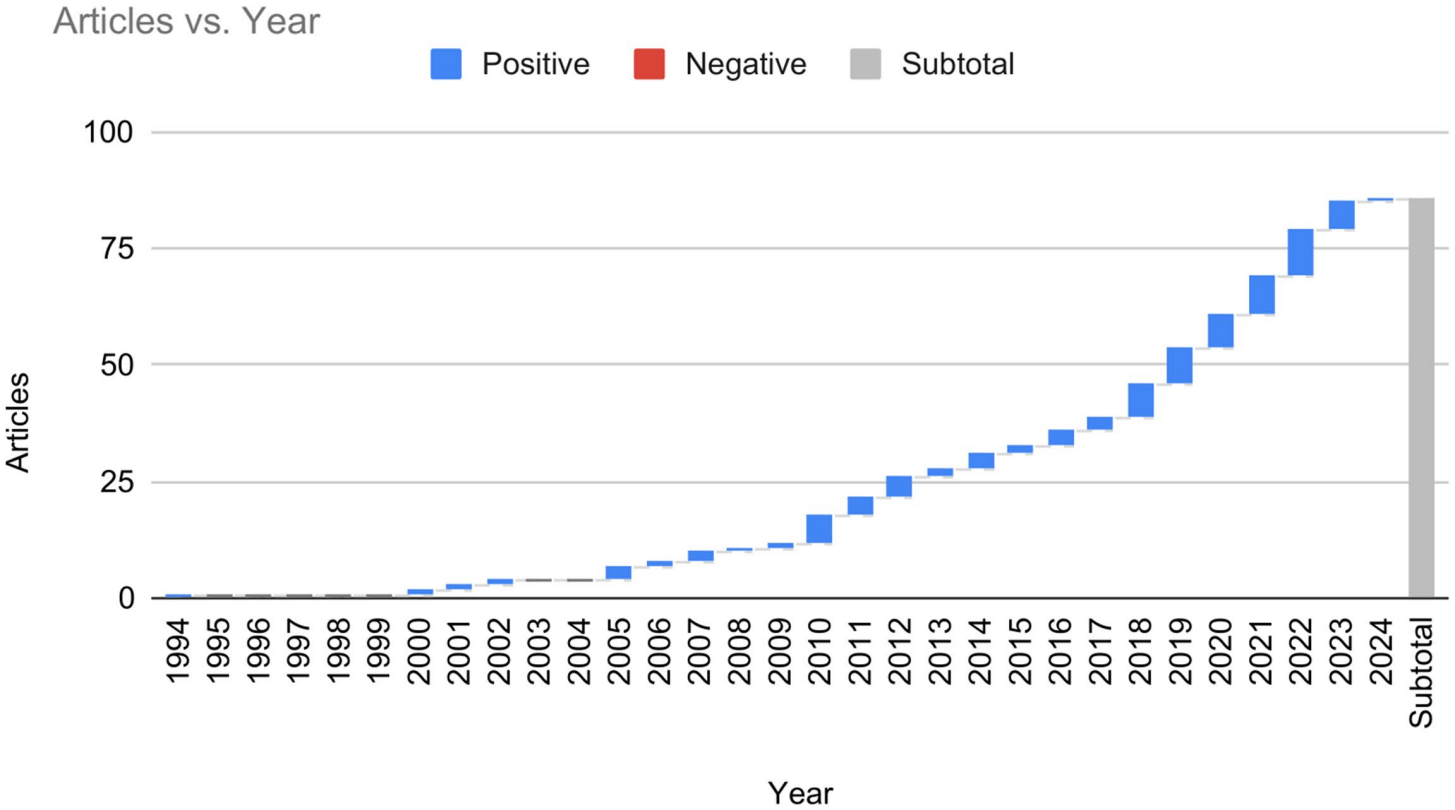
Year-wise publication of bibliometric papers. This figure represents the publication trend of bibliometric papers between 1994 and 2024. The data was retrieved from the Scopus database.

Within 30 years, a total number of 94 documents have been published. The first publication was recorded in the year 1994. After that, no publications were recorded in the SCOPUS database till 2000. Only one document was published thereafter till 2002. This waxing and waning trend continued till 2009. This year onwards the number of publications has increased steadily with 2020 marking the publication of 12 documents, the highest number of papers to date. This indicates that the research interest in this topic is increasing steadily. This also indicates that researchers are yet to synthesize MI and turn it into an applied concept, a concept that has the potential to explain everyday moral behavior (Figure 2).

Moral Intelligence is being investigated by various scholars from different disciplines. This is evident from the analysis of subject areas. The total number of publications can be classified into the following categories: Social Sciences (37; 23.9%), Medicine (23; 14.9%), Arts and Humanities (24; 15.6%), Business and Management (12; 7.8%), Nursing (13; 8.4%), Computer Science and Engineering (18; 11.6%), Psychology (8; 5.2%), Economics and Econometrics and Finance(6; 3.9%), Health Professions (3; 1.9%) and others (10; 6.5%). This shows that Moral Intelligence is indeed receiving attention from various disciplines. Social sciences and arts and humanities, arts and humanities, and medicine have the highest number of publications, while disciplines like business and management and computer science are taking an interest in developing and implementing this concept. Psychology, a discipline that tries to integrate behavior, emotion and cognition has not yet taken cognizance of the potential of MI as it accounts for only 5.2% of the total publications. It is also interesting to note that MI receives interest from various disciplines because it has an interdisciplinary appeal which can open new horizons for research collaborations. Results show that the publications were published in 4 different languages. The most common language is English (82; 88.17%) followed by Persian (7; 7.52%), German (2; 2.15%), Korean (1; 1.07%) and Slovenian (1; 1.07%).

### Preferred journals

Our analysis shows that Moral Intelligence is slowly gaining the interest of researchers. The most relevant source analysis in R identified six journals with at least two articles published on the topic of this paper. Of these eight journals, four are published by Universities and Institutes. The journals included are Acta Bioethica published biannually by the Interdisciplinary Center for Studies in Bioethics of the University of Chile, Salmand: Iranian Journal of Aging published by the University of Social Welfare and Rehabilitation Sciences, Opcion published by Universidad del Zulia and Journal of Mazandaran University of Medical Sciences published by Mazandaran University of Medical Sciences. The other five journals include Advances of Environmental Biology published by the American Eurasian Network of Scientific Information, European Journal of Educational Research published by the Eurasian Society of Education, Research and Perspectives in Psychiatric Care published by Wiley and Ethik in der Medizin published by Springer Nature.

The source impact was analyzed by the Temporal-Community Based Index (TC Based Index). The top 10 most impactful sources are Ethics and Information Technology (68), Journal of Business Ethics (61), Journal of Moral Education (34), Library of Ethics and Applied Philosophy (34), New Directions for Child and Adolescent Development (34), Management Science Letter (31), Management Research Review (27), Applied Artificial Intelligence (21), Criminal Justice and Behavior (15) and European Journal of Economics, Finance and Administrative Sciences (14).

Cite score is an alternative to Impact Factor and helps in understanding the impact of the journals based on the citation of articles of that journal. In the above table, it can be seen that the cite score of all the journals is relatively low. The decision to publish an article in a journal can be based on the cite score as well as the reach of the journal to the target audience. The low cite score of a few of the above journals may be attributed to the use of a language other than English. Also, we can see one journal has been discontinued from Scopus since 2020. All of these factors may have influenced the low cite score of the journals. So, fellow researchers are advised to choose the journal for publication keeping in mind the points mentioned above.

### Leading countries, top institutions and international collaboration

**Figure 3:**
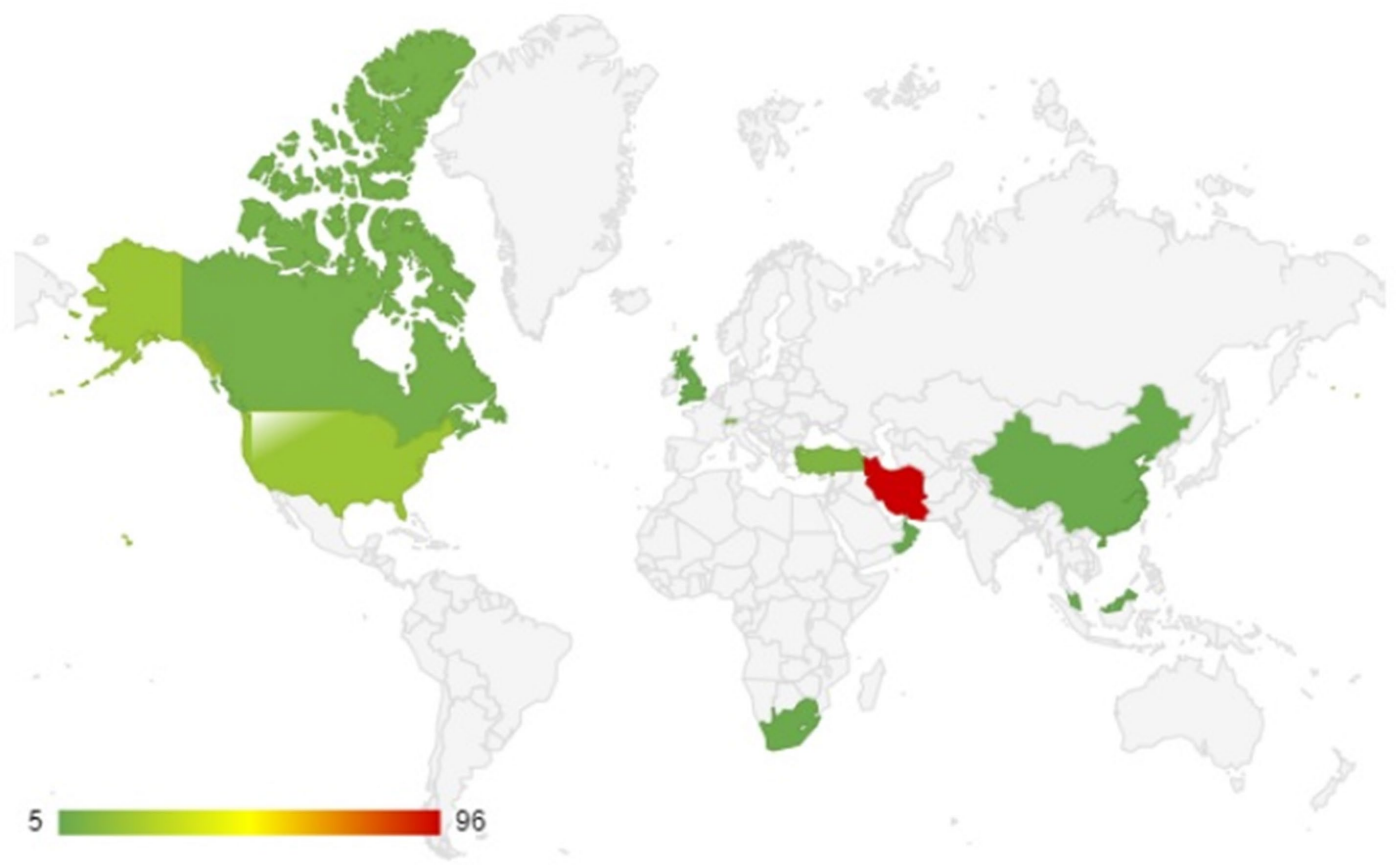
Number of publications of the top 15 countries on moral intelligence.

Figure 3 shows the top 15 countries having publications on Moral Intelligence. The two countries with the most publications are Iran and the United States and these two combined account for 53.16% of the total country-wise publication (Table 2). Iran alone published 27 articles which accounts for 34.17% of the total publication. While the United States ranks two in the list with 15 publications, the number of publications is less than half of Iran and it contributes about 18.98% of the publication. Germany, Switzerland and Turkey all have five publications each on the list and together they account for 18.98% of the total publications. Canada, China, Romania and the UK have three publications each and they account for 15.18% of the total publications. It is evident from this analysis that Iran has the highest number of researchers working on Moral Intelligence.

**Table 2:** The top six most productive journals on MI research with their most cited article.

	Journal	TP	TC	Cite score (2023)	The most cited article	Times cited	Publisher
1	Journal of Mazandaran University of Medical Sciences	3	13	0.6	Relationship between ethical intelligence and professional behavior in nurses	9	Mazandaran University of Medical Sciences
2	Research and Perspectives in Psychiatric Care	2	6	3.4	Reflection of nurses’ moral intelligence levels on care behaviors	4	WILEY
3	Opcion	2	0	NA	The relationship between moral intelligence and emotional intelligence with life satisfaction	0	Universidad del Zulia
4	Iranian Journal of Aging	2	4	1.9	Factors Related to Nurses’ Attitudes Toward the Elderly Care: The Predictive Roles of Altruism, Moral Intelligence, Life Satisfaction and General Health	4	University of Social Welfare and Rehabilitation Science
5	European Journal of Educational Researach	2	7	3	Teachers’ moral intelligence: A scale adaptation into Turkish and preliminary evidence	6	Eurasian Society of Educational Research
6	Ethik in der Medizin	2	4	1.1	Moral enhancement through neurosurgery? Feasibility and ethical justifiability	3	Springer Nature
7	Advances in Environmental Biology	2	3	N/A	The relationship between moral intelligence and academic progress of students Third year of high school course in Tabriz city	2	American-Eurasian Network for Scientific Information
8	Acta Bioethica	2	4	0.4	New concept in clinical care: Proposal of a moral intelligence scale	4	Interdisciplinary Center for Studies in Bioethics of the University of Chile

TP, total publications; TC, total citations.

**Figure 4:**
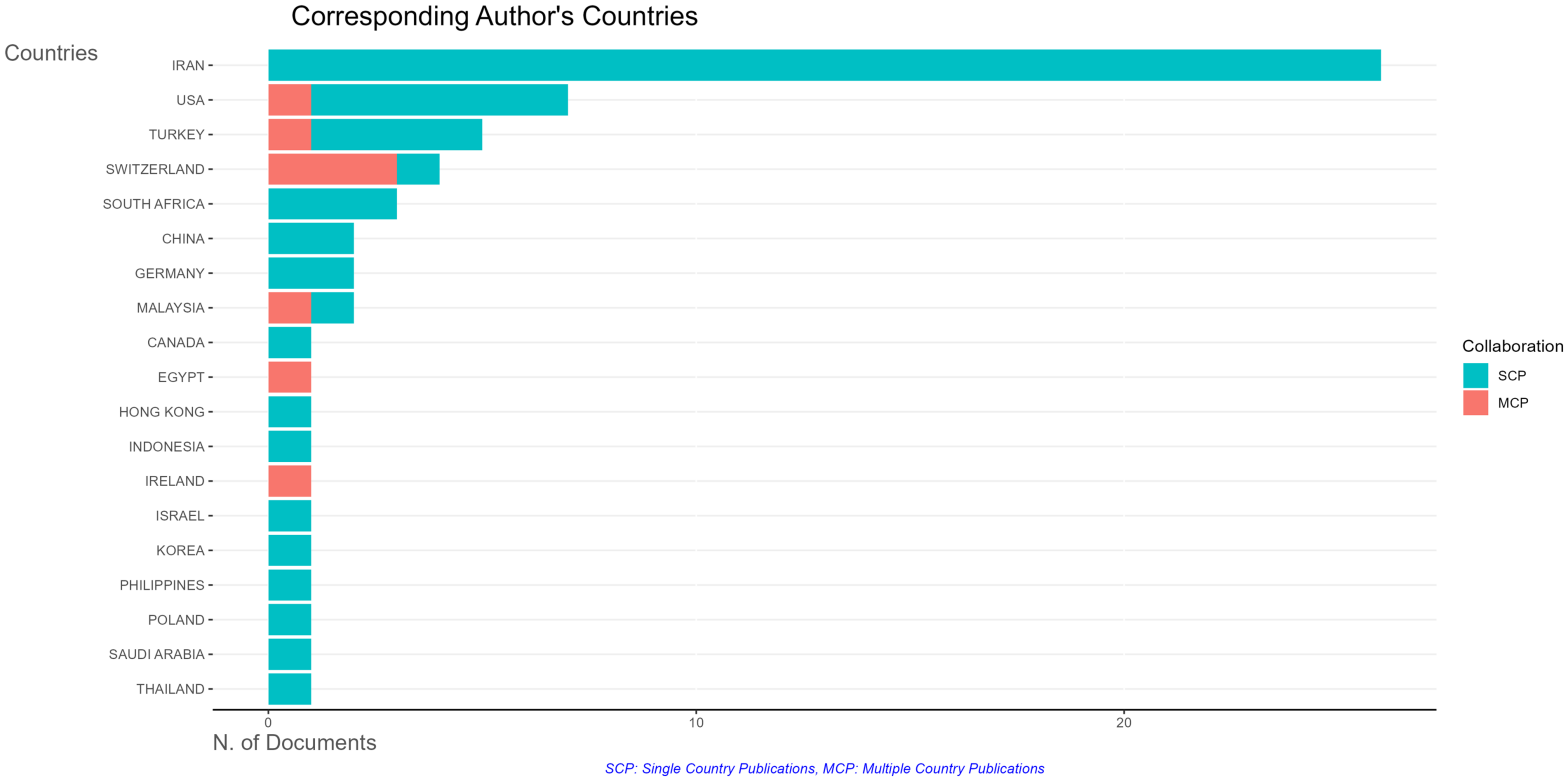
Countries with MCP on moral intelligence are highlighted in the plot.

In addition, Figure 4 shows the countries involved in Multi-Country Publication which is indicative of inter-country collaboration. The total number of MCP is eight. Out of these eight, the highest number of MCP can be found in Switzerland (30, 37.5%) while the United States, Turkey, Malaysia, Egypt and Slovenia have one MCP each. Since the topic is still in its nascent stage the number of collaborations is also on the lower side. International collaborations help researchers to develop skills and new perspectives on a topic which further helps to develop a discipline. Also, this is helpful for budding researchers to gain repute as a researcher in their respective fields of study.

### Leading authors

Table 3 clearly shows that the authors cluster around two countries. Out of the 10 authors, six authors are from Iran, and four authors are from Switzerland. The publications range from 2014 to 2021 which demonstrates that this topic has started gaining ground only recently. The role of co-authorship has been mentioned in the above table. The last position is usually assumed to be a supervisory role, although there are no guidelines mentioning that. Accordingly, the above authorship should be understood (Table 4).

**Table 3:** The top six countries with the most productive academic institutes/universities with multi-country publications.

Rank	Country	TPc	MCP	Most productive academic institute	TPi
1	Switzerland	4	3	Universität Zürich	4
2	United States	14	1	Yale University	2
3	Turkey	5	1	Eskişehir Osmangazi Üniversitesi	2
4	Malaysia	2	1	Universiti Malaysia Terengganu	1
5	Egypt	1	1	American University in Cairo	1
6	Slovenia	2	1	Slavistično društvo Slovenije	1

TPc, total publications of a given country; TPi, total publications of a given academic institution; MCP, multi-country publications.

**Table 4:** List of the 10 most prolific authors in MI research area.

	Author	Scopus author ID	Year of 1st publication	Total publication on MI	TP	h-Index	TC	Current affiliation	Country
1	Christen, Markus	56,275,127,800	2014b	4	111	18	1,528	Universität Zürich	Switzerland
2	Tanner, Carmen	7,202,846,947	2014b	4	34	15	1,637	Universität Zürich	Switzerland
3	Katsarov, Johannes	57,192,197,076	2020c	3	9	5	75	Universität Zürich	Switzerland
4	Nobahar, Monir	6,506,747,614	2020b	3	54	10	240	Semnan University of Medical Sciences and Health Services	Iran
5	Raiesdana,Nayyereh	36,696,968,400	2020c	3	14	6	126	Semnan University of Medical sciences and Health Services	Iran
6	Schmocker, David	57,201,739,315	2019a	3	4	3	33	Universität Zürich	Switzerland
7	Ghorbani, Maryam	57,226,774,587	2021a	2	2	1	1	Sabzevar University of Medical Sciences	Iran
8	Leili, Ehsan Kazemnezhad	36,182,721,200	2018c	2	244	18	1,252	Social Determinants of Health Research Center (GUMS)	Iran
9	Mahdavifar, Neda	57,113,721,900	2021c	2	25	7	377	Sabzevar University of Medical Sciences	Iran
10	Majidi, Seyed Ali	57,204,623,702	2020b	2	5	1	7	slamic Azad University, Rasht Branch, Rasht	Iran

*Role in co − authorship, superscripts. ^a^First author. ^b^Co-author. ^c^Last author.

Christen Markus and Carmen Tanner, both affiliated with Universität Zürich, led the list with a total publication of four documents each. The first document on Moral intelligence by both researchers was published in 2014. Christen Markus has a total publication of 111 documents and an h-index of 18. Carmen Tanner has a total publication of 33 documents with an h-index of 18. The 3rd prolific author Johannes Kastarov on this topic is also affiliated with Universität Zürich with three documents on Moral Intelligence. The first document was published in 2020. He has a total publication of nine documents with an h-index of 5. The last author from Universität Zürich is Schmocker, David ranked 6th on the list with three publications on Moral Intelligence and a total publication of four documents with an h-index of 3.

Monir Nobahar (h-index = 10) and Nayyereh Raisedana (h-index = 6) ranked 4 and 5th, respectively, are affiliated with Semnan University of Medical Sciences and Health Services have collaborated on three articles related to Moral Intelligence.

Maryam Ghorbani (h-index = 1), is 7th ranked, Neda Mahdavifar (h-index = 6) is ranked 9th are affiliated with Sabzevar University of Medical Sciences. Both of them have published two documents on Moral Intelligence and the first publication for all the authors is in 2021. There are no other collaborating authors found from the above list.

It can be concluded that most of the publications on MI are being conducted by authors affiliated with Universität Zürich, Semnan University of Medical sciences and Health Service and Sabzevar University of Medical Sciences. While the authors affiliated with Universität Zürich are interested in the conceptualization of Moral Intelligence as a concept (Tanner and Christen, 2014) and the development of business ethics and moral sensitivity through the use of a moral game (Tanner et al., 2022), the authors from Sabzevar University of Medical Sciences focused on nurses and the predictive power of Moral Intelligence among other variables (Mohammadi et al., 2020). Lastly, authors from Semnan University of Medical sciences and Health Service are working on variables related to Moral Intelligence which includes self-compassion, cultural competence, social capital and job satisfaction.

### Author keyword

#### Terminology and concept

Our result showed the initial number of author keywords was 210. After filtering synonymous and cogeneric words a total of 197 keywords were analyzed. Out of these 173 keywords, 173 (86.93%) were only used once, 19 (9.54%) were used twice, 5 (2.51%) were used thrice and 2 (1%) keywords met the threshold of 4 occurrences. Since we were interested in highlighting the upcoming areas of research related to Moral Intelligence we set the minimum threshold of one occurrence for the keyword network mapping in VOSviewer.

Our result showed that the most frequent keyword was Moral intelligence which is not a surprise as the keyword for the search was MI. The other keyword that had the maximum threshold of four occurrences was that of nurse (5 occurrences, 11 links), highlighting that most studies are being conducted on the nurse population. We also came across terms like moral sensitivity (3 occurrences, 11 links), ethics (3 occurrences, 10 links), ethical leadership (3 occurrences, 13 links) and forgiveness (3 occurrences, 14 links), which occurred 3 times. Traits like altruism, care, compassion, integrity, and responsibility occurred twice in the current database. Other terms included moral psychology, elderly, job satisfaction, clinical ethics and moral competence.

#### Emerging topics

**Figure 5:**
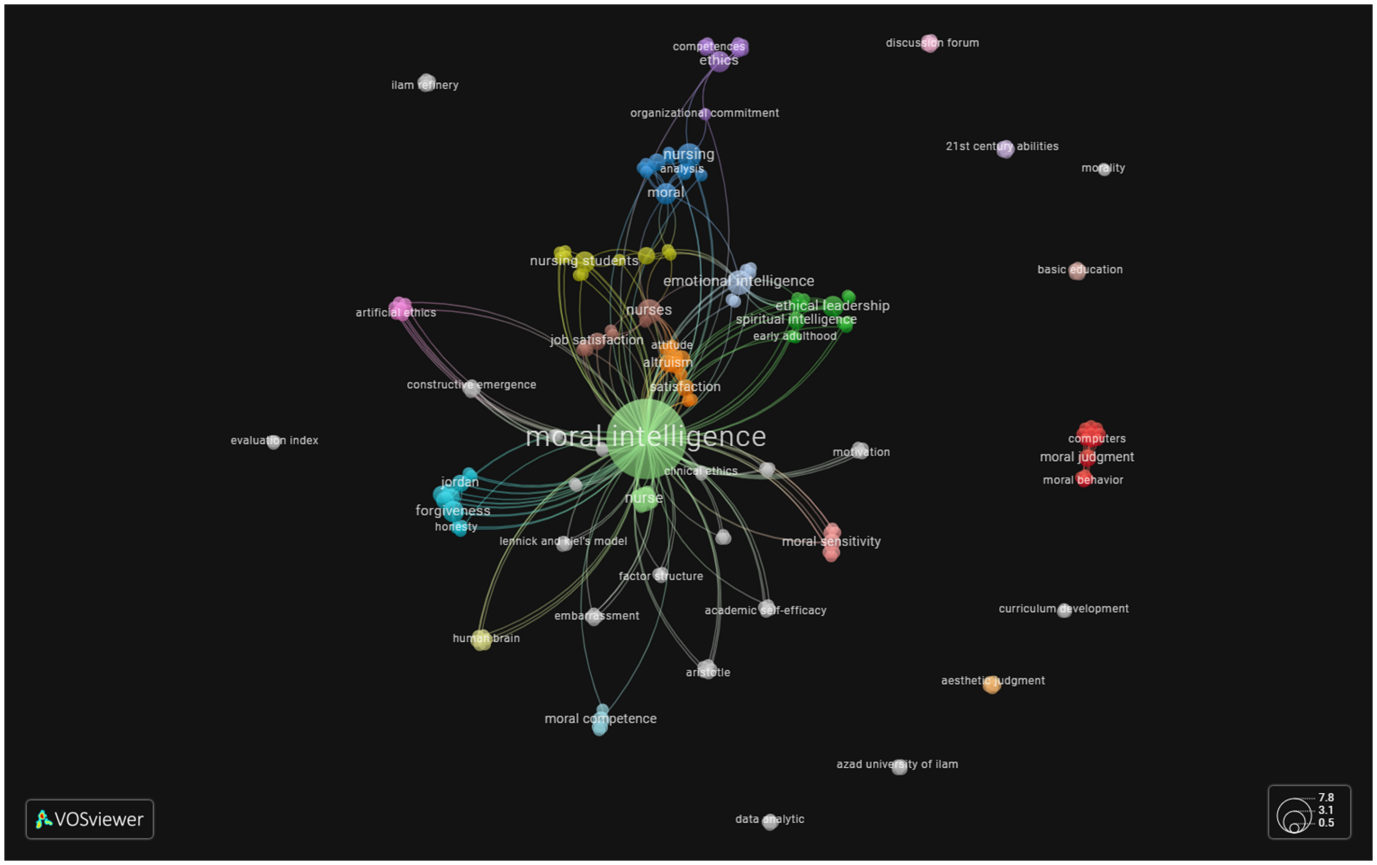
A screenshot of the bibliometric map created based on author keywords co-occurrence with overlay visualization mode. Minimum occurrences of a keyword are set to one. The following URL can be used to open this figure in VOSviewer: https://tinyurl.com/25r82xo6.

Emerging topics were defined as those with only one occurrence. One of the primary aims of this paper is to highlight the upcoming areas of investigation for researchers who are interested in Moral Intelligence research. These include decision-making, machine ethics, moral agent, artificial ethics, co-evolution of human and artificial moral agents, green purchase intention, ethical education, moral sensitivity, organizational citizenship behavior, organizational identity, social capital, social values, and Organizational commitment (Figure 5).

#### Significance and practical implications of moral intelligence

It is important to mention here that morality is not analogous to Moral Intelligence. Morality is related to moral principles that an individual inculcates since childhood through time or social interaction during one’s lifetime. Researchers like Lennick and Kiel (2005) defined MI as the application of universal moral standards to one’s values, goals and actions. However, the scope of Moral Intelligence is yet to be fully understood. MI can be further elaborated as the ability to safeguard one’s moral standards while navigating difficult life situations. In this section, a brief description of the scope of application of MI in various disciplines has been discussed. It has been observed that MI has been explored in various branches of psychology which is discussed below.

#### Organizational decision making

MI has its potential at an organizational level. Studies have demonstrated that MI influences organizational citizenship behavior which is mediated by principled leadership and trust in leaders (Engelbrecht and Hendrikz, 2020). Individuals with high moral intelligence may not fall into restrictive moral standards. A morally intelligent person will try to maximize the profitability or the desired outcome while efficiently safeguarding one’s moral principles. In this regard, it may be assumed that the recruitment of individuals with high levels of moral intelligence will be beneficial for an organization. According to Lennick and Kiel (2005), who utilized a trait-based approach to moral intelligence, the most effective strategy for building a successful business is to employ individuals with the highest moral and ethical standards. Emphasis on moral intelligence is considered to be even more crucial than emotional intelligence.

Positions requiring decision-making might benefit more from a person who has a high MI. More research can yield the dimensions of MI and people falling into these identified dimensions may excel in certain job categories. For example, people scoring higher on consequentialism may be better decision-makers when it comes to finding a middle way between safeguarding the organization’s profitability and one’s moral value. Hence performing better in those positions, thus demonstrating the importance of MI in practical organizational settings.

#### MI and education policy

MI, like other types of intelligence, is not inherent and is learned through nurturing, teaching, training, and modeling (i.e., an ethics-observing social environment is essential in moral development; Mohammadi et al., 2020). It has been demonstrated that levels of moral sensitivity increase following a holistic teaching approach, as opposed to discipline-specific (Zarifsanaiey et al., 2016). Tanner and Christen (2014) in their research outlined the various dimensions of Moral Intelligence. One of the dimensions was Moral Sensitivity. Tanner et al. (2022), in an experimental study, evaluated the effectiveness of a serious moral game, to see the effects of playing a game with moral situations and improving moral sensitivity.

Clarken (2010) emphasized the importance of training children in dealing with moral aspects of life. The moral aspect of education has often been overlooked by policymakers leaving the children to decide on moral situations based on their learnings from mass media, social media and peers. Without a guiding force, the child becomes confused and may engage in actions that are detrimental to the child and others. Training in MI will not only inculcate important moral values in the child but also train them on how to effectively manage a moral situation while safeguarding their values, reducing the chances of moral injury.

### Application of MI in artificial intelligence

Recent research highlights the integration of Moral Intelligence (MI) in AI systems. Cunneen et al. (2019) examined how AI in automated vehicles can make moral decisions during unavoidable accidents. They emphasized the need to integrate MI with laws, ethical theories, human values, and human rights to guide AI decision-making, using the trolley dilemma to explore the differences between human and AI driving intelligence.

Ji and Rau (2018) proposed a classification system for voice intelligent agents (VIAs) based on moral intelligence, showing that interaction methods significantly impact classification results. They recommend text interaction for more accurate MI assessment, revealing the complex relationship between interaction complexity, task completion time, and evaluator satisfaction. Additionally, Ji and Rau discussed the challenges of implementing MI in AI, arguing that AI needs human-like attributes such as consciousness, theory of mind, social skills, and empathy to navigate morally significant situations effectively. This complexity necessitates specialized tests or highlights the limitations of current tests in evaluating AI’s moral intelligence.

These studies underscore the importance of MI in AI, highlighting its practical implications for developing AI systems capable of making safe, legal, and appropriate decisions in complex moral contexts (Cunneen et al., 2019; Ji and Rau, 2018).

### Measurement of MI

The corpus of literature used in this study revealed that the most frequently used tool to measure MI is the Moral Competency Inventory developed by Lennick and Kiel (2005). According to the authors of the scale Moral Intelligence consists of traits like integrity, responsibility, compassion, and forgiveness. This scale has been used in the majority of the studies. Two more scales have been identified measuring MI. The first is the Moral Intelligence Scale for Healthcare Professionals developed by Mohammadi et al. (2024)). This scale includes three domains *viz.*, moral sensitivity, moral commitment and moral courage. The second scale has been developed by Ozturk et al. (2019) named Moral Intelligence Scale. It includes the dimensions of empathy, conscience, self-control, respect, kindness, tolerance, and fairness. However, no ability-based scale to measure MI has been identified.

### Limitations of study

The search strategy was restricted to the word ‘Moral Intelligence’. Hence the different components that make up Moral Intelligence have not been accounted for. This was done with the primary objective of capturing publications based on Moral Intelligence as the components that make up the construct are still in development.

Scopus had also identified 191 more documents on Moral Intelligence but they were not included as according to SCOPUS those documents that are classified as Secondary have incomplete or incorrect data or retrieved from the reference list of the journal articles or missing contents. Additional databases like the Web of Science can be searched for a comprehensive review.

### Scope and future directions

This study offers significant guidance to moral psychology researchers who are interested in Moral Intelligence. This will act as a quick reference guide for researchers. It seems there is a dearth of literature summarizing the research trends in Moral Intelligence. Hence, this paper will provide directions to researchers and help them in selecting topics related to MI. This paper demonstrates that the knowledge of MI is still fragmented and requires a structure. A clear theoretical framework will help researchers conceptualize and refine the concept. So, we encourage researchers to work toward theory development on MI.

Quantitative measurement is another important aspect that will help further the concept’s applicability. Researchers are encouraged to validate the robustness of the trait-based approach to Moral Intelligence. It remains elusive whether this concept is culturally based or transcends culture. Researchers need to work on defining the concept more clearly.

This paper also aims to stimulate discussion on whether MI as a concept can be applied to organizational settings more specifically for recruitment purposes. Different job positions call for different types of Moral intelligence. Understanding the moral demand of the job and selecting personnel accordingly will lead to better work performance. There is a need for understanding MI in terms of ability and trait; as has been adopted for Emotional Intelligence (O’Connor et al., 2019). Research on this aspect will cater to different job role needs.

MI as a concept is theoretically related to moral injury as has been highlighted in this paper. Although initially moral injury research was initially conceptualized for individuals engaged in combat settings (Litz et al., 2009) recent studies have demonstrated that police, journalists and veterinarians are also susceptible to moral injury (Williamson et al., 2018). The prevalence of moral injury apart from the above-mentioned occupations needs to be explored. It will also be interesting to see whether Moral Intelligence can act as a protective factor against moral injury. As MI can be seen as the ability to safeguard one’s moral values when faced with a moral situation it is assumed that those with higher levels of MI will have a lower prevalence of moral injury.

MI as a concept is in its infancy and researchers can explore the endless possibilities attached to it. The most important aspect of a new concept is its applicability and usefulness in practical life settings. Throughout the paper, we have tried to demonstrate the potential of MI to become an integral part of life.

## Conclusion

This study provided an overview of Moral Intelligence research trends based on 94 documents. There has been a steady rise in publications since 2009 and this trend is expected to continue. We discovered that countries like Iran, the United States and Switzerland have a substantial number of publications on this topic. The growth trend for Multi-Country Publications (MCP) is lower than expected. It seems that a lot of work has been done on the nurse population. Moral sensitivity, ethics, ethical leadership and forgiveness are emerging topics to be explored more. Lastly, we also reviewed the implications of Moral intelligence at an individual, societal and organizational level and discussed the considerations needed for developing tools to measure Moral Intelligence and ended with future directions for researchers interested in this domain of moral psychology.

## Data Availability

The original contributions presented in the study are included in the article/Supplementary material, further inquiries can be directed to the corresponding author.
